# Integration of Ultrasound into the Development of Plant-Based Protein Hydrolysate and Its Bio-Stimulatory Effect for Growth of Wheat Grain Seedlings In Vivo

**DOI:** 10.3390/plants10071319

**Published:** 2021-06-28

**Authors:** Karolina Trakselyte-Rupsiene, Grazina Juodeikiene, Darius Cernauskas, Elena Bartkiene, Dovile Klupsaite, Daiva Zadeike, Joana Bendoraitiene, Jonas Damasius, Jonas Ignatavicius, Sidona Sikorskaite-Gudziuniene

**Affiliations:** 1 Department of Food Science and Technology, Faculty of Chemical Technology, Kaunas University of Technology, 50254 Kaunas, Lithuania; karolina.trakselyte-rupsiene@ktu.edu (K.T.-R.); daiva.zadeike@ktu.lt (D.Z.); jonas.damasius@ktu.lt (J.D.); 2 Food Institute, Kaunas University of Technology, 50254 Kaunas, Lithuania; darius.cernauskas@ktu.lt; 3 Institute of Animal Rearing Technologies, Faculty of Animal Sciences, Lithuanian University of Health Sciences, 44307 Kaunas, Lithuania; elena.bartkiene@lsmuni.lt (E.B.); dovile.klupsaite@lsmuni.lt (D.K.); 4 Department of Food Safety and Quality, Faculty of Veterinary Medicine, Lithuanian University of Health Sciences, 44307 Kaunas, Lithuania; 5 Department of Polymer Chemistry and Technology, Faculty of Chemical Technology, Kaunas University of Technology, 50254 Kaunas, Lithuania; joana.bendoraitiene@ktu.lt; 6 JSC Nando, 53341 Kaunas, Lithuania; jonas@nando.lt (J.I.); sidona@nando.lt (S.S.-G.)

**Keywords:** plant biostimulants, plant-based protein hydrolysate, corn steep liquor, ultrasound treatment, enzymatic treatment, free amino acids

## Abstract

This study was dedicated to increasing the efficiency of producing plant-based protein hydrolysate using traditional and non-traditional treatments. Low- and high frequency ultrasound (US) at different intensities were applied to corn steep liquor (CSL) at 50 °C for 30 min, and enzymatic hydrolysis was performed using industrially produced alkaline protease. The efficiency of US and enzymatic treatments was characterized by protein solubility (soluble protein (SP) content, hydrolyzed protein (HP) concentration, and free amino acid (FAA) profile) and kinetic parameters: Michaelis–Menten constant (KM) and apparent breakdown rate constant (kA). A significant effect of 37 kHz US pre-treatment for CSL enzymatic hydrolysis was found and resulted in the highest HP concentration (17.5 g/L) using the lowest enzyme concentration (2.1 g/L) and the shortest hydrolysis time (60 min). By using US pre-treatment, on average, a 2.2 times higher FAA content could be achieved compared to traditional hydrolysis. Additionally, results for the kinetic parameters k*_M_* and k*_A_* confirmed the potential of applying US treatment before hydrolysis. The effect of CSL protein hydrolysate on plant growth was tested in vivo on wheat grain seed germination and resulted in the significant increase in germination parameters compared to the control treatment. These findings indicate that by-products of starch industry could be a promising source for the production of low-cost sustainable biostimulants.

## 1. Introduction

Current trends in EU agriculture are very strongly focused on reducing negative environmental impacts, forcing the search for the effective use of renewable sources in the development of novel biostimulant formulations to improve crop yield in sustainable agriculture [1]. Seed “priming” is a common technique and can lead to a faster and more uniform seed germination process. By-products from wet corn milling, such as corn steep liquor (CSL), are optimal nitrogen feedstocks relevant for the development of new biostimulants. One major group of plant biostimulants is protein hydrolysates, defined as a mixture of peptides and amino acids [2].

One traditional option for processing protein hydrolysates is the use of biocatalysts to transform these residual feedstocks into high-value target protein hydrolysates. Microbial proteases such as Alcalase, Protamex, and Flavourzyme were used to hydrolyze corn gluten meal [3]. Amino acids are important for the central metabolism of seeds. Primarily, they are used for the synthesis of seed-storage proteins; they are also used as precursors for the production of secondary metabolites and as an energy source [4]. Natural biostimulants (e.g., Terra-Sorb^®^ complex), obtained via enzymatic hydrolysis, are characterized by a high (20%) content of free amino acids: aliphatic amino acids (Ala, Val, Gly, Pro, Ile, Leu), hydroxy-amino acids (Ser, Thr), sulphur-containing amino acids (Cys, Met), aromatic amino acids (Trp, Phe, Tyr), acidic amino acids (Asp, Glu), and basic amino acids (His, Arg, Lys) [5]. A number of studies confirmed that biostimulants based on amino acid have a significant impact on plant growth in winter wheat [6], lettuce [7], and soybean [8].

The main disadvantages of traditional hydrolysis are long duration and a low degree of hydrolysis (DH). The majority of studies were attributed to develop methods to improve the substrate conversion efficiency and reduce the hydrolysis time [9]. Ultrasound treatment attracted much attention as a novel physical processing technology to improve the extraction process of bioactive components [10] as well as assisting enzymatic hydrolysis [9,11]. Some researchers reported that ultrasound treatment significantly improves the bioactivity of selected products, the substrate conversion efficiency, and the rate of reaction. One of the most important factors affecting the efficiency of sonochemical reactions is the ultrasound frequency [12].

This study hypothesized whether ultrasound treatment of biomass could increase the efficiency of enzymatic hydrolysis. The effects of ultrasound pre-treatment at different frequencies (37 and 850 kHz) and different intensities on the protein hydrolysis of CSL were investigated. Furthermore, the effects of enzymatic pre-treatment on the kinetic parameters of CSL enzymolysis were determined. The biostimulatory effect of the obtained plant-based protein hydrolysate was tested on wheat seed germination characteristics.

## 2. Materials and Methods

### 2.1. Corn Processing By-Products and Their Analysis

The CSL, extracted from corn during the steeping process (44.4% crude protein in dry matter), was provided (Nando, Lithuania) in a frozen state, and all samples were stored in a freezer (−18 °C).

AACC-approved Methods 44–15.02 and 46–12.01 [13] were applied to determine the moisture and the total nitrogen content, respectively. The nitrogen content was multiplied by a factor of 6.25 to calculate the crude protein content. A polarimetric method [14] was used to measure starch content. The chemical composition of CSL is presented in Table 1. All the basic chemicals used in this study were obtained from JSC Eurochemicals (Vilnius, Lithuania).

### 2.2. CSL Ultrasound Pre-Treatment Procedure

CSL pre-treatment was performed using two types of ultrasonication: (i) low frequency (37 kHz) and high intensity and (ii) high frequency (850 kHz) and low intensity. Ultrasound (US) treatment was performed using the same experimental equipment as described by Bartkiene et al. [15] and Juodeikiene et al. [16]. Each 20 g sample of CSL at pH 9.0 (adjusted by 6 M NaOH) was treated in both cases for 30 min at 50 °C, and each experiment was performed in triplicate.

The efficiency of CSL US treatment was characterized according to the soluble proteins determined by the Bradford assay using bovine serum albumin (BSA) as standard [17]. A standard curve was made of BSA (0.0, 0.2, 0.4, 0.6, 0.8, 1.0, and 1.5 mg/mL), and absorbance was read at 595 nm. All determinations were conducted in triplicate.

### 2.3. Enzymatic Treatment of CSL

Concentrated liquid alkaline protease was selected for the CSL protein hydrolysis. The enzyme preparation with an activity of 400,000 U/g was obtained from AB Baltic Enzymes (Vilnius, Lithuania). The hydrolysis experiment was carried out at various substrate (21–210 g protein/L) and enzyme concentrations (1.05–42.00 g/L) at different pH values (7.0 and 9.0) at 70 °C for 120 min. During hydrolysis, the pH was continuously maintained by addition of 6 M NaOH. The enzymes were inactivated at 90 °C for 15 min.

In order to describe the kinetics of CSL hydrolysis, DH according to the pH-stat method, hydrolyzed protein (HP) concentration, and initial reaction rate (*V*_0_) as well as the kinetic parameters K*_M_* and k*_A_* were determined using Equations (1)–(3), respectively [12]:

(1) $$DH\left(\%\right)=\frac{h}{h_{tot}}=\frac{N_{b}\times B\times 100}{\propto \times M_{p}\times h_{tot}}$$

where: *N_b_* and *B* are concentration (mol/L) and volume consumed (mL), respectively, of NaOH; α is the average degree of dissociation of the α-NH_2_ groups; *M_p_* is the mass of protein to be hydrolyzed (g); and *h_tot_* is the total number of peptide bonds in the protein substrate (which is 9.2 mmol/g for corn protein).

(2) $$C_{t}=C_{0}\times DH\times 0.01,$$

where: *C*_0_ and *C_t_* are initial and hydrolyzed protein concentration (g/L), respectively; and *DH* is the degree of hydrolysis.

(3) $$\frac{1}{V_{0}}=\frac{K_{M}}{k_{A}E_{0}}\times \frac{1}{C_{0}}+\frac{1}{k_{A}E_{0}},$$

where: *V*_0_ is the initial reaction rate (g/L/min); K*_M_* is the Michaelis–Menten constant (g/L); k*_A_* is an average value of the apparent breakdown rate constant (1/min), representing the binding frequency between substrate and enzyme; *E*_0_ is the enzyme concentration (g/L); and *C*_0_ is the substrate concentration (g/L). The slope and the intercept of the line 1/*V*_0_ against 1/*C*_0_ are K*_M_*/(k*_A_E*_0_) and 1/(k*_A_E*_0_), respectively.

### 2.4. Extraction and Determination of Free Amino Acids

CSL protein hydrolysates were frozen at −18 °C. Lyophilization was performed using pilot scale sublimator/freeze-dryer Freeze Drying Plant Sublimator 3 × 4 × 5 (Zirbus Technology, Bad Grund/Harz, Germany). Samples were dehydrated under vacuum at −40 °C for 24 h. Lyophilized CSL protein hydrolysates were used to determine free amino acids (FAA). FAA extraction was carried out using 0.1 M HCl as solvent; the sample: solvent ratio was 1:7.5. Samples were mixed on a magnetic stirrer for 60 min. After extraction, 5.0 mL of 6% sulphosalicylic acid was added to precipitate the proteins. Samples were centrifuged (6000 rpm, 14 min, 12 °C). After that, the supernatant was used to determine FAA by ultrafast liquid chromatography (UFLC) with automated o-phthalaldehyde (OPA)/9-fluorenylmethyl chloroformate (FMOC) using the same experimental conditions as described by Jukonyte et al. [18]. Each sample was analyzed in triplicate.

### 2.5. Biostimulatory Effect of CSL on Wheat Seed Treatment and Germination

The biostimulatory effect of CSL was evaluated using wheat grains (*Triticum aestivum*), which were provided by JSC, KG Group. Seeds were not subjected to fungicide, insecticide, or other treatments; all samples were stored in dry and dark conditions (10–12 °C). Prior to treatment with CSL samples, the grain seeds were surface-sterilized with 2% sodium hypochlorite for 15 min and then washed thoroughly six times with autoclaved distilled water. Sterilized seeds were added to plastic bags, and different CSL formulations (10% suspension of each sample CSL/CSL1/CSL2/CSL3) were added at a concentration of 100 µL/g seeds. Tested seeds were soaked for 30 min; for the control sample, seeds were soaked with distilled water. The biostimulatory effect of CSL was evaluated by a seed germination study.

The seed germination experiment was carried out using the roll method according to Dapkevicius et al. [19]. Seeds coated with different CSL preparations were dried at room temperature before germination. Test and control wheat seeds (100 seeds, four replicates) were placed evenly (1.5 cm apart) on wet Whatman filter No. 1 filter paper (20 cm wide and 100 cm long). The top of the seeds was covered with a wet strip of filter paper (8 cm wide). The strips were rolled into stiff rolls which were soaked in water in a separate bowl. For germination of seeds, after 3 days of treatment, seeds were analyzed, and their germination percentage (GP) was calculated using the Equation (4):

(4) $$\mathrm{GP}\;=\;\frac{Number\;of\;seeds\;germinated}{Total\;number\;of\;seeds\;sown\;for\;germination\;}\times 100$$

After 7 days of treatment, stem and root length were evaluated.

The fresh and the dry mass of roots and stems were quantified by weighing on a precision scale. The stems and the roots were dried in an oven at 60 °C for 24 h [20].

### 2.6. Statistical Analysis

All analyses were performed at least in triplicate (*n* = 3) and were expressed as the average ± standard deviation. Results were analyzed via Kruskal–Wallis one-way ANOVA test and Tukey’s test using statistical package SPSS for Windows XP version 15.0 (SPSS Inc., Chicago, IL, USA, 2007). In order to quantify the strength of the relationship between the variables, a linear Pearson’s correlation was determined. Differences were considered statistically significant when *p* < 0.05.

## 3. Results and Discussion

### 3.1. Effect of CSL Ultrasonication on the Enzymatic Treatment

**Figure 1:**
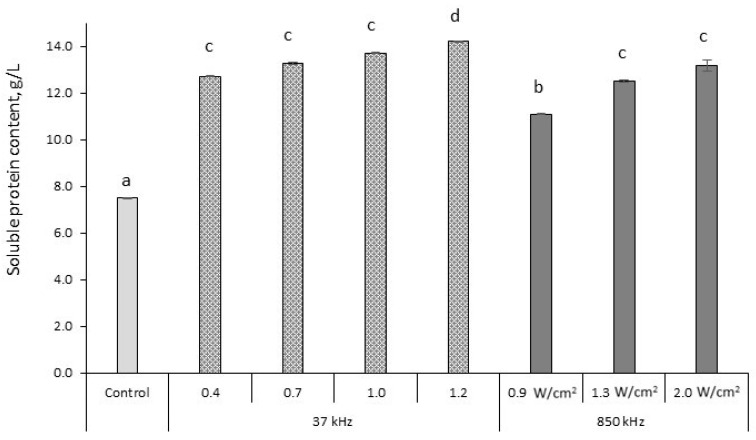
Changes in soluble protein content in corn steep liquor (CSL) samples after low- (37 kHz) and high frequency (850 kHz) ultrasound treatment at different intensities (40%, 70%, 100%, and 120%–37 kHz and 0.9, 1.3, and 2.0 W/cm^2^–850 kHz) for 30 min at 50 °C, pH 9.0. Data expressed as a mean value (*n* = 3) ± SD; SD—standard deviation; a–d means with different letters are significantly different (*p* < 0.05).

Alternative technological means for increasing the efficiency of protein solubilization and obtaining a higher FAA yield from by-products are still being sought. Recently, considerable interest arose in the application of new physical methods such as US for the pre-treatment of plant biomasses. The effect of the ultrasonication on chemical composition and functional properties under different conditions is an essential point in developing novel biostimulants and improving the production process. In this study, the application of low- and high frequency US to CSL from corn processing was taken into consideration. Analysis of the distribution of soluble protein within ultrasonicated samples using low- and high frequency US at different intensities is presented in Figure 1.

The study showed that US power at low- as well as at high-frequency significantly affected the solubility of the protein in CSL. US treatment at 37 and 850 kHz increased the soluble protein (SP) content by 79.7% and 63.4%, respectively, compared to the control sample (7.5 g/L) (Figure 1). The highest CSL protein solubility (14.2 g/L) was obtained using low frequency US (37 kHz) at 120% power (increased by 89.3% compared to the untreated sample). Using lower power US (40%, 70%, and 100%), a less obvious effect of treatment was obtained than at 120% and SP content increased by 69.9%, 77.2%, and 82.8%, respectively, compared to the control.

Most studies focused on US treatment at low frequency (20 kHz) and high intensity for plant proteins and their impact on structural changes as well as physical and functional properties of the proteins. The results obtained in this study are in agreement with the literature: low frequency (20 kHz) US cavitation affects the structure of proteins by destroying molecular bonds such as hydrogen bonds and electrostatic forces. These changes improve the solubility and other physical and chemical characteristics of proteins. According to Li et al. [21], the highest solubility of rapeseed protein (16.71 mg/mL, compared to other preparations) can be obtained by US treatment at a fixed frequency of 20 kHz. This might be due to the increase in interactions between protein and water generated by ultrasonic cavitation, which results in the formation of small fragments of proteins and promotes protein solubility.

The obtained results showing that the application of high frequency (850 kHz) US is efficient for the solubilization of the proteins in CSL (Figure 1) are in agreement with those presented by Juodeikiene et al. [16] showing that high frequency (850 kHz) and low power (0.9 w/cm^2^, 1.3 w/cm^2^) ultrasonication significantly increase flaxseed protein solubility (on average by 16.2%–22.4% compared to the control sample). In this study, a significant effect of the intensity used on the SP content was observed for this treatment. The highest solubility of CSL proteins (13.2 g/L) was detected after exposure to 2.0 W/cm^2^ US (850 kHz); when using lower US intensities (0.9 and 1.3 W/cm^2^), the changes in protein solubility, compared to the control, were less obvious (11.1 and 12.5 g/L, respectively).

During the next stage, US treatment (37 kHz at 120% power and 850 kHz at 2.0 W/cm^2^ exposure) of CSL before enzymatic hydrolysis was taken into consideration. The kinetics of CSL enzymolysis were determined in order to describe the efficiency of US pre-treatment.

**Figure 2:**
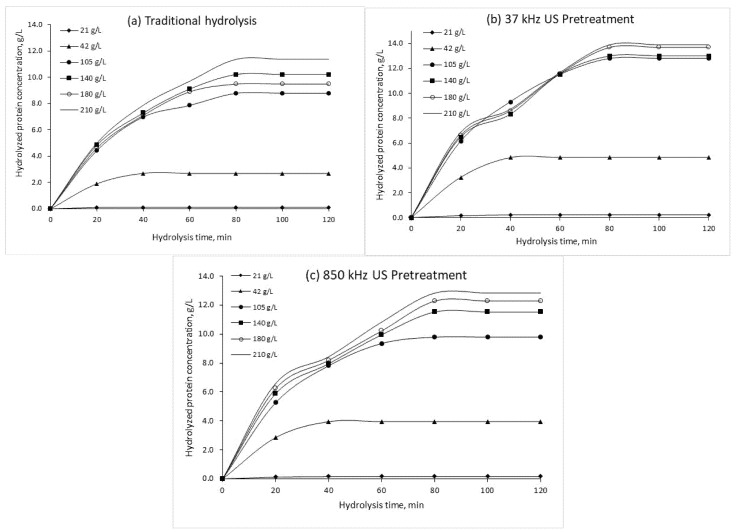
Hydrolyzed protein concentrations obtained by traditional hydrolysis (**a**), 37 kHz US pre-treated hydrolysis (**b**) and 850 kHz pre-treated hydrolysis (**c**) at different protein concentrations (*E*_0_ = 1.05 g/L, pH = 9.0, T = 70 °C). US—ultrasound treatment.

**Figure 3:**
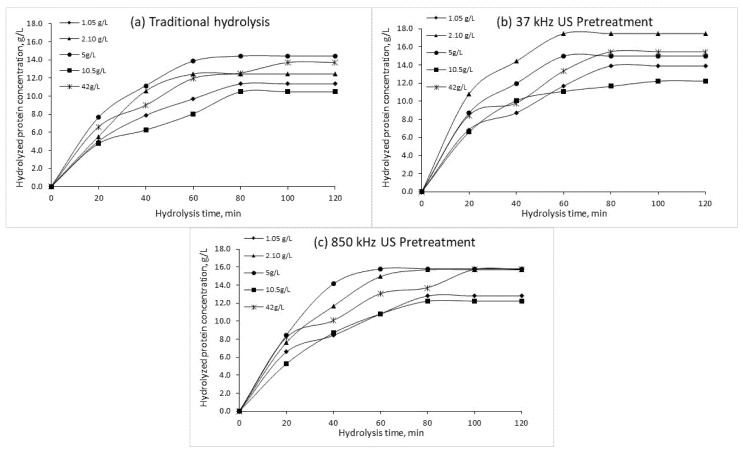
Hydrolyzed protein concentrations obtained by traditional hydrolysis (**a**), 37 kHz US pre-treated hydrolysis (**b**) and 850 kHz pre-treated hydrolysis (**c**) at different enzyme concentrations (*C*_0_ = 210 g/L, pH = 9.0, T = 70 °C). US—ultrasound treatment.

**Figure 4:**
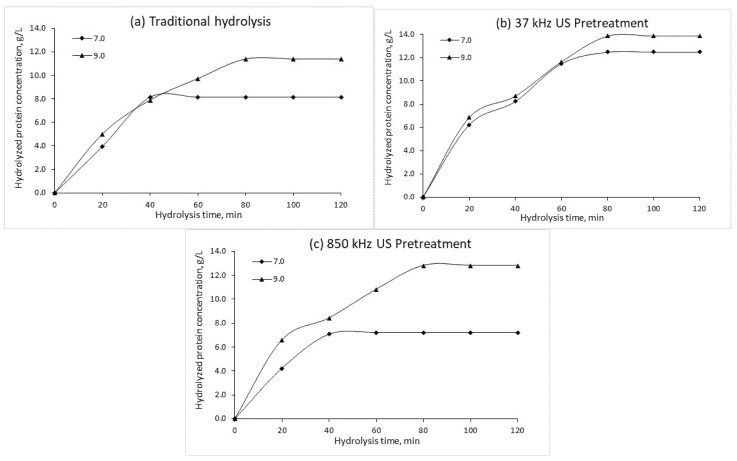
Hydrolyzed protein concentrations obtained by traditional hydrolysis (**a**), 37 kHz US pre-treated hydrolysis (**b**) and 850 kHz pre-treated hydrolysis (**c**) at different pH (*C*_0_ = 210 g/L, *E*_0_ = 1.05 g/L, T = 70 °C). US—ultrasound treatment.

Additional analysis was performed to test the effects of low and high frequency US pretreatment for enzymatic hydrolysis of CSL (duration of 120 min) using alkaline protease preparations and their effect on HP content. Figure 2, Figure 3 and Figure 4 show the HP concentrations during traditional and US pre-treated hydrolysis (120 min) at different protein concentrations, enzyme concentrations, and pH.

A Kruskal–Wallis H test showed that there was a statistically significant difference in hydrolyzed protein content between the different samples at certain time intervals (*p* < 0.05). The study showed (Figure 2) that US pre-treatment enhanced the hydrolysis rate, especially at higher protein concentrations (105, 140, 180, and 210 g/L). At the highest protein concentration (210 g/L), the application of low frequency (37 kHz) US was more efficient than high frequency (850 kHz) US treatment and resulted in an increase in HP concentration after the first 20 min (36.8% and 31.6%, respectively) compared to traditional hydrolysis (5.01 g/L). Furthermore, the duration of enzymatic exposure to CSL proteins was taken into consideration, and the highest HP content (13.9 and 12.82 g/L, respectively) was obtained after 80 min compared to the control sample (11.37 g/L).

In the perspective to determine the optimal conditions for enzymatic treatment, different enzyme concentrations were tested, and an increase in HP content was noted in all tested samples (Figure 3). Using traditional hydrolysis, the maximum HP concentration (14.4 g/L) was obtained after 80 min using 5 g/L of enzyme. High frequency (850 kHz) US pre-treatment accelerated hydrolysis, and the maximum HP content (15.8 g/L) was achieved after 60 min using 5 g/L of the enzyme. The results indicate that 37 kHz US pre-treatment remarkably improved the enzymatic hydrolysis process. Application of low frequency US before enzymatic hydrolysis resulted in the highest HP concentration (17.5 g/L) using the lowest enzyme concentration (2.1 g/L) and the shortest hydrolysis time (60 min).

The HP concentrations obtained by traditional and US-treated enzymatic hydrolysis at different pH are shown in Figure 4. HP concentrations increased with increasing pH for both traditional and US-treated hydrolysis. In all cases, the highest HP concentrations were obtained at pH 9.0, and US treatment increased the final HP concentration on average by 17.3%. However, analysis of HP changes at pH 7.0 revealed a contrary effect of high frequency (850 kHz) US treatment, which resulted in a decrease of the final HP concentration on average by 11.5%. On the other hand, the final HP concentration at pH 7.0 obtained with low frequency (37 kHz) US pre-treatment represented a 53.1% increase.

Analysis of the application of alkaline enzymes into CSL media confirmed that the concentration of the enzyme preparation as well as the treatment time itself play key roles in the HP content. Direct addition of the selected enzyme preparation to raw materials is a highlight in the treatment of sustainable CSL, helping to manage the overall standardization of the hydrolysis process and the quality of the final product. This study proved that US treatment using a low frequency (37 kHz) at 120% power could be an alternative approach for increasing protein solubility.

Many researchers noted that protein properties can be improved by US treatment [22], enhancing enzyme activity [9,23] and extracting polysaccharides [24]. According to Jin et al. [12], MPU pre-treatment remarkably improves the enzymolysis of CGM, and this technology could be applied to produce peptides in the protein proteolysis industry.

The results for kinetic parameters confirm that ultrasonic pre-treatment can accelerate enzymatic hydrolysis: the initial hydrolysis rate (*V*_0_, g/L) increased on average by 36.2% with ultrasonic treatment (Table 2).

The Michaelis–Menten constant (K*_M_*) indicates the affinity of an enzyme for a given substrate: the lower the K*_M_* value is, the higher is the affinity of the enzyme for the substrate. A major decrease of K*_M_*, on average by 33.5%, was noted for the enzymatic hydrolysis of CSL with 37 kHz US pre-treatment. The decrease in K*_M_* revealed that US pre-treatment improved the affinity between enzyme and substrate. Additionally, a 30% increase of k*_A_* value was observed comparing low frequency US-assisted CSL hydrolysis with the traditional process. The results are in agreement with the findings of Jin et al. [12] who reported that enzymolysis of CGM can be improved by MPU pre-treatment: it decreased K*_M_* by 26.1% and increased k*_A_* by 7.3% compared to traditional hydrolysis.

During the next stage, the FAA profiles were investigated in order to better understand the roles of different treatments during processing as well as to identify particular amino acids that may be relevant for using processed CSL samples as biostimulants. CSL hydrolysates CSL1, CSL2, and CSL3 with the highest HP content (traditional hydrolysis–14.4 g/mL, 37 kHz pre-treated hydrolysis–17.5 g/mL and 850 kHz, pre-treated hydrolysis–15.8 g/mL) were selected for FAA determination and for coating wheat grain seeds.

### 3.2. Influence of Ultrasonic Pre-Treatment for Enzymatic Hydrolysis of CSL on FAA Profile

At this stage, the influence of US at different frequencies (37 and 850 kHz) on FAA content was compared. Table 3 shows the FAA composition (g/100 mL CSL) of SP after hydrolysis of CSL with alkaline protease and in combination with US pre-treatment at different frequencies.

The FAA composition of the SP in CSL before the treatment was essentially identical to that of CSL [25,26] with a dominance of nonpolar FAA such as alanine (1.39), isoleucine (0.91), proline (0.82), tryptophan (0.40), and phenylalanine (0.27) and sulphur-containing FAA such as methionine (0.32) and cysteine (0.21). However, the protein content as well as the FAA profile of co-products obtained during corn processing differed depending on the corn anatomical parts entering them during fractionation.

Prolamins (known as zein) are globular storage proteins found mainly in maize (the equivalent to wheat gliadin and barley hordein). Almost all the zein (45%–50% of the total proteins) with a high proportion of hydrophobic amino acids (proline and glutamine) is present in the endosperm, while glutelin is present between the endosperm and the germ [27,28]. The albumin and globulin fractions are found mostly in the germ [28]. Among the protein-based by-products of corn wet milling, CGM contains the endosperm proteins, while corn gluten feed (CGF) and CSL contain the germ proteins. Xiao et al. [26] identified FAA and their derivatives as potential markers of CSL and showed that the fingerprinting technique and PSA analysis can be effective tools to evaluate the quality of CSL. This paper focused on the alkali-soluble proteins in CSL and their hydrolysis into FAA for biostimulant production.

The results show that positive changes in the total amount of FAA were obtained as well as changes in separate FAA. The application of US for the treatment of CSL resulted in a higher FAA yield, which has a relationship with HP content (r = 0.943). The following tendencies for the production of FAA upon applying US and enzymatic hydrolysis can be stated: (i) 37 kHz US pre-treatment of CSL increased the total FAA content up to 2.3 times, while an increase of up to 2.15 times was measured in the samples treated with 850 kHz as compared to the reference samples; (ii) traditional enzymatic hydrolysis of CSL was found to have a slight difference in FAA yield (an increase of up to 1.6 times was measured).

Relatively different trends in FAA yield were noticed with respect to particular US treatments. A positive effect of traditional enzymatic treatment of CSL on Asp, Ser, Gly, Thr, Ala, Tyr, Cys, Val, Met, Trp, Phe, Ile, Leu, Lys, and Pro was noticed, while ultrasonication (in both cases) exclusively encouraged the enhancement of all FAA. This observation suggests better conditions for FAA production using US pre-treatment before enzymatic hydrolysis. A comparable tendency of an increase of Asp, Glu, Ser, His, Gly, Thr, Arg, Ala, Tyr, Val, Met, Trp, Phe, Ile, Leu, and Pro in CSL was noticed when using 37 kHz US. In the case of 850 kHz US, higher Cys and Lys contents in CSL were noted.

The obtained results are in agreement with previous studies: initial FAA and those obtained by acidic hydrolysis in CSL samples from different sources (Russia, France, Great Britain, Italy, Germany, and Egypt) were almost the same. Alanine was found to be the predominant FAA in CSL. High levels of FAA such as glutamic and aspartic acids, serine, glycine, leucine, lysine, and valine were detected. Only in Italian and German dried CSL were lower amounts of phenylalanine, methionine, tyrosine, proline, and cysteine found [25]. According to Bartkiene et al. [15], ultrasonication is the most effective treatment for obtaining an increase of FAA content in bovine colostrum (BC). In this study, low frequency US treatment (37 kHz, 160 W) increased the amounts of Val, Leu, Ile, Thr, and Met in the samples. The relationship between the amino acid constituents after different CSL treatments and their biostimulatory effect on wheat seeds were taken into consideration.

### 3.3. The Impact of CSL Protein Hydrolysate Application on Wheat Grain Seedlings Germination

In the present study, we examined the effect of new products—protein hydrolysates—based on amino acids produced by US treatment and enzymatic hydrolysis of CSL (at a dose of 100 µL/g seeds) on the germination process of winter wheat. The biostimulatory effect of CSL hydrolysates compared to reference samples (CSL—without additional treatment; control—seeds treated with water) was tested in laboratory trials on wheat seed germination, and results are shown in Table 4.

The application of CSL protein hydrolysates (CSL, CSL1, CSL2, CSL3) increased GP on average by 9.4% compared with that in the seeds not treated with CSL (control). During the experiment, significant effects on wheat seed root length and stem length were observed based on amino acids (CSL1, CSL2, and CSL3) and the reference sample (CSL) and the control. A positive effect of applying the preparations on the germination characteristics of wheat seeds (root length and stem length) was observed compared to the control groups (CSL and control): for CSL1, root length and stem length were 9.76 and 6.32 cm and 15.1% and 17.5% higher, respectively; for CSL2 (37 kHz pre-treatment), they were 11.03 and 7.20 cm and 30.1% and 33.8% higher, respectively; and for CSL3 (850 kHz pre-treatment), they were 10.14 and 6.90 cm and 19.6% and 28.3% higher, respectively. Similar tendencies were observed for the effect of protein hydrolysates on root and stem fresh and dry weight. Treatment with biostimulants resulted in a higher biomass content of wheat stems and roots. CSL protein hydrolysates (CSL1, CSL2, and CSL3) increased the fresh weight of wheat grain roots and stems on average by 21.3% and 27.1%, respectively, as well as dry weight.

In this study, it was found that the roles of FAA change are different according to the seed state. Comparing the germination parameters of wheat samples with the CSL used to treat them, a strong positive correlation was obtained between root length, stem length, and total FAA in CSL (r = 0.925 and 0.977, respectively). In the case of FAA, the strongest effect on seed growth (root length, stem length) was shown for valine (r = 0.942 and 0.983, respectively), while between these parameters and other FAA, r varied from 0.737 to 0.983. Most FAA (Asp, Ser, Thr, Arg, Tyr, Cys, Val, Trp, Phe, Ile, Leu, Lys, Pro) had a positive effect on root development as well as on stem growth. However, some of the FAA (Glu, Gly, Ala, Met) showed a different, better effect on stem development compared to that on root growth.

Amino acids play an important role in plants. In their review, Amir et al. [4] presented unique functional roles during seed development of FAA from the Asp-family pathway (Lys, Met, and Thr) and branched-chain amino acids (BCAAS) (Val, Ile, and Leu) as well as Pro. For example, BCAAS and the metabolism of BCAAS contribute to seed development and germination versus other tissues [29]. Methionine affects the levels of storage compounds [30]. Proline (Pro) plays an important role in embryo development [31]. 

Based on this study, it can be assumed that wheat seed germination and growth can be improved by the application of plant-based protein hydrolysate, which provides nutrients such as FAA and soluble peptides necessary for seed development. Popko et al. [6] also reported a positive effect of protein hydrolysate (applied in soil and foliar treatments) on growth and yield of winter wheat crops. Similar results were presented by Colla et al. [32], who tested the effect of plant-derived protein hydrolysate containing amino acids and small peptides and found that this product enhances nitrogen uptake, stimulates growth, and elicits a hormone-like activity (auxin and gibberellin-like activity) in corn, tomato, and dwarf pea.

## 4. Conclusions

CSL is produced during corn starch production, and 44.4% of the protein (in d.m.) remains in this by-product. It is an inexpensive potential source of nitrogen for producing protein hydrolysates by biochemical industries. Our study on the application of low and high frequency US treatment before enzymatic hydrolysis to produce biostimulants enriched with FAA shows that CSL sonication could be an effective way to accelerate the hydrolysis process and enhance protein solubility.

US treatment of CSL using different frequencies (37 and 850 kHz) resulted in a change in protein fraction by increasing the formation of SP on average by 71.6%. When combining US with hydrolysis, 37 kHz US pre-treatment led to the highest HP concentration (17.5 g/L) using the lowest enzyme concentration (2.1 g/L) and the shortest hydrolysis time (60 min). Additionally, a major decrease of K*_M_* on average by 33.5% as well as a 30.0% increase of k*_A_* were obtained with low frequency US pre-treatment. These findings confirm that US application has a positive effect on CSL hydrolysis. Moreover, 37 kHz sonication of CSL resulted in the greatest increase (2.3 times) in FAA content, while traditional hydrolysis was less effective and resulted in an increase of FAA by 1.6 times.

Positive results were obtained by applying protein hydrolysates for coating and germination of wheat grain seeds. A significant increase of root and stem length was measured when applying sonicated and enzymatically hydrolyzed products as a wheat grain seed coating. Finally, this study confirmed that US can be applied for biotransformation of CSL to protein hydrolysate. The bio-process enlarges the functionality of protein solubilization and application of the modified by-products for seed germination improvement. The application of US is a sustainable way to include non-traditional methods of by-product valorization to produce bioactive compounds in the agriculture industry. Future research is needed to evaluate the effects of plant treatment with protein hydrolysates on the development of biologically active components in plants.

## Figures and Tables

**Table 1:** Chemical composition of corn steep liquor (% d. m.).

Component	Concentrations
Dry matter (%)	51.7 ± 0.29
Crude protein (%)	44.4 ± 0.57
Starch (%)	1.3 ± 0.02

Data expressed as a mean value (*n* = 3) ± SD.

**Table 2:** Kinetic parameters k*_M_*, k*_A_* for traditional CSL hydrolysis and US pre-treated.

	V_0_ (g/L * min^−1^)	k*_M_*/(k*_A_E*_0_)	1/(k*_A_E*_0_)	k*_M_* (g/L)	k*_A_* (min^−1^)
Traditional hydrolysis	0.267 ± 0.01 ^a^	113.34 ± 0.27 ^a^	3.21 ± 0.07 ^a^	35.35 ± 0.91 ^a^	0.30 ± 0.005 ^a^
37 kHz pretreated hydrolysis	0.367 ± 0.01 ^b^	57.63 ± 0.23 ^c^	2.45 ± 0.03 ^b^	23.50 ± 0.40 ^c^	0.39 ± 0.002 ^b^
850 kHz pretreated hydrolysis	0.360 ± 0.01 ^b^	66.26 ± 0.34 ^b^	2.59 ± 0.04 ^b^	28.74 ± 0.38 ^b^	0.37 ± 0.004 ^b^

Data expressed as a mean value (*n* = 3) ± SD; SD—standard deviation. ^a–c^ Means within a column with different superscript letters are significantly different (*p* < 0.05); *V*_0_—initial hydrolysis rate, K*_M_*—the Michaelis–Menten constant, k*_A_*—apparent breakdown rate constant, *E*_0_—enzyme concentration.

**Table 3:** The effect of ultrasound pre-treatment at low and high frequencies (37 kHz and 850 kHz) for enzymatic hydrolysis of CSL on free amino acid composition.

Free Amino Acid, g/100 mL CSL	CSL	CSL1	CSL2	CSL3
Aspartic Acid	0.04 ± 0.01 ^a^	0.09 ± 0.04 ^a^	0.12 ± 0.06 ^a^	0.11 ± 0.06 ^a^
Glutamic acid	0.11 ± 0.11 ^a^	0.13 ± 0.02 ^a^	0.18 ± 0.04 ^a^	0.17 ± 0.03 ^a^
Serine	0.28 ± 0.03 ^a^	0.43 ± 0.05 ^a^	0.59 ± 0.12 ^b^	0.55 ± 0.05 ^b^
Histidine	0.04 ± 0.05 ^a^	0.03 ± 0.05 ^a^	0.04 ± 0.07 ^a^	0.04 ± 0.07 ^a^
Glycine	0.19 ± 0.02 ^a^	0.24 ± 0.02 ^a^	0.33 ± 0.06 ^c^	0.31 ± 0.01 ^b^
Threonine	0.25 ± 0.01 ^a^	0.39 ± 0.05 ^a^	0.51 ± 0.11 ^c^	0.52 ± 0.05 ^b^
Arginine	0.01 ± 0.02 ^a^	0.06 ± 0.06 ^a^	0.09 ± 0.09 ^a^	0.09 ± 0.08 ^a^
Alanine	1.39 ± 0.02 ^a^	1.84 ± 0.32 ^a^	2.58 ± 0.60 ^c^	2.41 ± 0.37 ^b^
Tyrosine	0.02 ± 0.01 ^a^	0.07 ± 0.05 ^a^	0.09 ± 0.07 ^a^	0.09 ± 0.07 ^a^
Cysteine	0.21 ± 0.03 ^a^	0.32 ± 0.03 ^b^	0.49 ± 0.04 ^c^	0.50 ± 0.04 ^c^
Valine	0.34 ± 0.11 ^a^	0.62 ± 0.02 ^b^	0.89 ± 0.12 ^c^	0.83 ± 0.04 ^c^
Methionine	0.32 ± 0.08 ^a^	0.45 ± 0.05 ^a^	0.62 ± 0.20 ^a^	0.61 ± 0.14 ^a^
Tryptophan	0.39 ± 0.06 ^a^	0.68 ± 0.09 ^b^	0.98 ± 0.17 ^c^	0.95 ± 0.04 ^c^
Phenylalanine	0.27 ± 0.02 ^a^	0.47 ± 0.20 ^a^	0.67 ± 0.16 ^b^	0.63 ± 0.10 ^b^
Isoleucine	0.91 ± 0.16 ^a^	1.36 ± 0.20 ^a^	1.96 ± 0.42 ^b^	1.89 ± 0.22 ^b^
Leucine	0.03 ± 0.05 ^a^	0.07 ± 0.04 ^a^	0.10 ± 0.06 ^a^	0.10 ± 0.06 ^a^
Lysine	0.06 ± 0.08 ^a^	0.12 ± 0.14 ^a^	0.15 ± 0.22 ^a^	0.15 ± 0.21 ^a^
Proline	0.82 ± 0.60 ^a^	1.99 ± 0.60 ^b^	2.46 ± 0.63 ^c^	2.23 ± 1.03 ^b^
Total FAA	5.67 ± 0.19 ^a^	9.34 ± 1.21 ^a^	12.87 ± 1.40 ^b^	12.18 ± 2.32 ^b^

CSL—untreated corn steep liquor. CSL1—corn steep liquor hydrolyzed with alkaline protease. CSL2—corn steep liquor pre-treated with 37 kHz US (120% intensity) and hydrolyzed with alkaline protease. CSL3—corn steep liquor pre-treated with 850 kHz US (2.0 W/cm^2^ intensity) and hydrolyzed with alkaline protease. Data expressed as a mean value (*n* = 3) ± SD; SD—standard deviation. ^a–c^ Means between columns with different superscript letters are significantly different (*p* < 0.05). FAA—free amino acid.

**Table 4:** Growth characteristics of wheat grains seeds using for the treatment of seeds different CSL hydrolysates versus control after 7 days germination.

	GP (%)	Root Length (cm)	Stem Length (cm)	Root Fresh Wt. (g)	Root Dry Wt. (g)	Stem Fresh Wt. (g)	Stem Dry Wt. (g)
Control	85.3 ± 0.58 ^a^	8.48 ± 0.52 ^a^	5.38 ± 0.49 ^a^	0.0115 ± 0.0002 ^a^	0.0558 ± 0.0005 ^a^	0.0053 ± 0.0005 ^a^	0.0055 ± 0.0027 ^a^
CSL	93.3 ± 1.15 ^b^	8.77 ± 0.69 ^a,b^	6.04 ± 0.74 ^a^	0.0165 ± 0.0004 ^b^	0.0650 ± 0.0004 ^a^	0.0066 ± 0.0006 ^b^	0.0063 ± 0.0004 ^a^
CSL1	90.7± 0.58 ^b^	9.76 ± 0.54 ^b,c^	6.32 ± 0.65 ^a^	0.0180 ± 0.0015 ^b,c^	0.0690 ± 0.0015 ^a,b^	0.0078 ± 0.0021^2^	0.0068 ± 0.0002 ^a^
CSL2	95.7± 0.58 ^c^	11.03 ± 0.47 ^c^	7.20 ± 1.22 ^a^	0.0195 ± 0.0005 ^c^	0.0732 ± 0.0002 ^b^	0.0083 ± 0.0004 ^c^	0.0071 ± 0.0002 ^b^
CSL3	92.3± 0.58 ^b^	10.14 ± 0.29 ^c^	6.90 ± 0.52 ^a^	0.0190 ± 0.0020 ^c^	0.0705 ± 0.0003 ^c^	0.0081 ± 0.0002 ^c^	0.0069 ± 0.0001 ^b^

Data expressed as a mean value (*n* = 3) ± SD; SD—standard deviation. ^a–c^ Means within a column with different superscript letters are significantly different (*p* < 0.05); CSL—untreated corn steep liquor; CSL1—corn steep liquor hydrolyzed with alkaline protease; CSL2—corn steep liquor pre-treated with 37 kHz US (120% intensity) and hydrolyzed with alkaline protease; CSL3—corn steep liquor pre-treated with 850 kHz US (2.0 W/cm^2^ intensity) and hydrolyzed with alkaline protease; PH—protein hydrolysate; GP—germination percentage.

## Data Availability

No new data were created or analyzed in this study. Data sharing is not applicable to this article.
